# The cGas–Sting Signaling Pathway Is Required for the Innate Immune Response Against Ectromelia Virus

**DOI:** 10.3389/fimmu.2018.01297

**Published:** 2018-06-14

**Authors:** Wen-Yu Cheng, Xiao-Bing He, Huai-Jie Jia, Guo-Hua Chen, Qi-Wang Jin, Zhao-Lin Long, Zhi-Zhong Jing

**Affiliations:** State Key Laboratory of Veterinary Etiological Biology, Key Laboratory of Veterinary Public Health of Agriculture Ministry, Lanzhou Veterinary Research Institute, Chinese Academy of Agricultural Sciences, Lanzhou, China

**Keywords:** innate immunity, cGas, Sting, type I interferon, ectromelia virus

## Abstract

Activation of the DNA-dependent innate immune pathway plays a pivotal role in the host defense against poxvirus. Cyclic GMP-AMP synthase (cGAS) is a key cytosolic DNA sensor that produces the cyclic dinucleotide cGMP-AMP (cGAMP) upon activation, which triggers stimulator of interferon genes (STING), leading to type I Interferons (IFNs) production and an antiviral response. Ectromelia virus (ECTV) has emerged as a valuable model for investigating the host–Orthopoxvirus relationship. However, the role of cGas–Sting pathway in response to ECTV is not clearly understood. Here, we showed that murine cells (L929 and RAW264.7) mount type I IFN responses to ECTV that are dependent upon cGas, Sting, TANK binding kinase 1 (Tbk1), and interferon regulatory factor 3 (Irf3) signaling. Disruption of cGas or Sting expression in mouse macrophages blocked the type I IFN production and facilitated ECTV replication. Consistently, mice deficient in cGas or Sting exhibited lower type I IFN levels and higher viral loads, and are more susceptible to mousepox. Collectively, our study indicates that the cGas–Sting pathway is critical for sensing of ECTV infection, inducing the type I IFN production, and controlling ECTV replication.

## Introduction

Innate immune responses to pathogen infection are initiated with the recognition of microbial pathogen-associated molecular patterns (PAMPs) through a limited number of germline-encoded receptors called pattern-recognition receptors (PRRs) (1–3). PAMPs represent conserved molecule motifs within a class of microbes that are recognized by cells of the innate immune system, which include lipopolysaccharides, peptidoglycans, or nucleic acids (RNA and DNA) (2–4). PRRs exist in the plasma or endosomal membranes, cytoplasm, and nucleus of some cell types to sense both extracellular and intracellular infections (5–7). Nucleic acid-sensing PRRs are one of the major subsets of PRRs that sense DNA and RNA (7–9). Some members of these membrane-bound PRRs, such as Toll-like receptors (TLR3, 7, 8, and 9), are located in the endosomes that detect environmental RNA or DNA, while others, such as the DNA sensors AIM2-like receptors (AIM2, IFI16, and IFIX) and cyclic GMP-AMP synthase (cGAS), and the RNA sensors RIG-I-like receptors (RIG-I, MDA5, and LPG2), recognize microbial nucleic acid in the cytosol and/or nucleus (10–13). Following the recognition of microbial RNA or DNA, the PRRs are activated through conformational changes or specific modifications that drive the induction of type I interferon (IFN) and pro-inflammatory cytokines to protect the host from the invading pathogens (14–16).

Currently, an increasing number of studies have suggested that the cGAS–stimulator of interferon genes (STING) pathway of cytosolic DNA sensing plays a major role in the immune defense against microbial pathogens (17). Upon recognition of DNA viruses, retroviruses, and intracellular bacteria, cGAS catalyzes the formation of the second messenger molecule cGMP-AMP (cGAMP), which in turn associates with and activates the STING (17–19). The activation of STING leads to its dimerization, relocalization, and prion-like aggregation, which then associates with TANK binding kinase 1 (TBK1) and causes the recruitment of interferon regulatory factor 3 (IRF3) (5). IRF3 phosphorylates and translocates to the nucleus where the production of type I IFNs is induced (19–21). Furthermore, it has been reported that STING acts as a direct sensor of cyclic dinucleotides such as mammalian 2′3′-cGAMP and prokaryotic 3′3′-cGAMP, c-di-GMP, c-di-AMP, which can also induce the production of type I IFNs (21, 22).

Poxviruses are large, enveloped, double-stranded DNA viruses that replicate entirely in the cytoplasm and cause human and veterinary diseases. The orthopoxvirus (OPV) genus of Poxviridae, including variola virus (VARV), vaccinia virus (VACV), monkeypox virus (MPXV), cowpox virus, and ectromelia virus (ECTV), cause acute infections in their target hosts. Despite the eradication of smallpox through a global vaccination campaign spearheaded by the World Health Organization in the late 1970s, other OPVs have been reported to persist in various animal species following natural or experimental infections (23). Currently, with the emergence of zoonotic MPXV, the outbreaks of VACV infections in dairy cattle and their transmission to humans, and cases of cowpox in humans, there is still great and essential significance as well as interest in the molecular mechanisms of poxvirus infections and/or protection from OPV infections (24, 25). ECTV, a mouse specific pathogen that causes mousepox, is closely related to VARV and has been used as a model for the study of the pathogenesis and immunobiology of OPV infection (26, 27).

Following ECTV infection, type I IFNs were shown to be induced *in vitro* and *in vivo* (28, 29). C57BL/6 mice deficient in *Ifnar1*, which encodes subunit 1 of IFNAR, an IFN α/β receptor, are highly susceptible to ECTV infections (28, 30, 31). Studies performed on murine cells and C57BL/6 mice have demonstrated that the induction of type I IFNs in draining lymph nodes (dLNs) during ECTV infections is because of the recognition of the virus indirectly by the TLR9–MyD88–interferon response factor 7 (IRF7) pathway and directly by the STING–IRF7/nuclear factor kappa B (NF-κB) pathway (32). Mice deficient in TLR9 and its adaptor protein MyD88 show higher viral loads, more serious pathology in the liver and spleen, and increased susceptible to ECTV infection than wild-type (WT) mice (31–34). Moreover, C57BL/6 mice deficient in the transcription factors IRF7 and NF-κB, which are downstream targets of both TLR9–MyD88 and STING, are also highly susceptible to mousepox (32). Notably, STING, as a critical adapter of the cytosolic DNA sensor, is also essential for the resistance to lethal ECTV infections as well as the expression of type I IFNs in the dLNs *in vivo* (32, 35). However, Dai (DNA-dependent activator of IFN-regulatory factors), a cytosolic DNA sensor upstream of STING, was demonstrated to be less important for resistance to mousepox (32). Therefore, the PRRs upstream of STING that contribute to the recognition of ECTV and resistance to mousepox remain unclear. In addition, cGAS has been recently discovered to be a general cytosolic DNA sensor upstream of STING that recognizes cytoplasmic DNA derived from a large spectrum of DNA viruses, retroviruses, bacteria, fungi, and parasites (4–7). Not surprisingly, VACV as DNA virus has the ability to trigger type I IFN production responses *via* cGas–Sting pathway (36). However, it is currently not known whether the cGAS is an important innate immune DNA sensor also for ECTV infection.

Here, we examine the contribution of the cGas–Sting pathway to the antiviral response to ECTV *in vitro* and *in vivo*. We showed that murine L929 and RAW264.7 cells, but not NIH3T3 cells, mount type I IFN responses against ECTV infection *via* the cGas–Sting pathway. Disruption of cGas and Sting expression by RNA interference or gene knockout impaired the expression of type I IFNs at the mRNA or protein levels induced by the virus. The induction of type I IFNs were abolished in *Tlr9^−/−^, cGas^−/−^, Sting^−/−^, Tbk1^−/−^*, and *Irf3^−/−^* macrophages (RAW264.7 and peritoneal macrophages) and had significantly increased viral titers in *cGas^−/−^* and *Sting^−/−^* RAW264.7 cells, respectively, compared with WT cells. We also demonstrate that ECTV infection triggered the phosphorylation of Tbk1 and Irf3. *In vivo*, mice deficient in *Tlr9, cGas*, or *Sting* blocked the production of type I IFNs and showed higher viral loads and serious pathology in the liver and spleen, and were more susceptible to lethality caused by infections with ECTV as compared with WT mice. Our results confirm that the cGas–Sting pathway is required for resistance to ECTV infections.

## Materials and Methods

### Cells and Virus

Vero (African green monkey kidney cell line), L929 (murine fibroblast cell line), NIH3T3 (murine embryo fibroblast cell line), and HEK293T cells were obtained from the China Center for Type Culture Collection, and were maintained in Dulbecco’s Modified Eagle’s Medium (DMEM, Hyclone) supplemented with 10% sterile fetal bovine serum (Gibco), 100 IU of penicillin/mL, and 100 µg/mL of streptomycin, and incubated at 37°C in the presence of 5% CO_2_. RAW-Lucia ISG (rawl-isg), RAW-Lucia ISG-KO-cGas (rawl-kocgas), RAW-Lucia ISG-KO-Sting (rawl-kostg), RAW-Lucia ISG-KO-Irf3 (rawl-koirf3), and RAW-Lucia ISG-KO-Tbk1 (rawl-kotbk) cells were purchased from the InvivoGen company and were grown in DMEM (Gibco) supplemented with 10% sterile fetal bovine serum (Gibco), 100 µg/mL of Normocin, and 200 µg/mL of Zeocin (InvivoGen).

The WT strain of ECTV was originally isolated from a naturally infected laboratory mouse and then propagated in Vero cells (37). Plaque-purified ECTV was serially passaged, and the virus titer was measured by plaque assays on Vero cells. UV-inactivated ECTV was irradiated under short-wave (254 nm) ultraviolet light for 2 h. The infectivity of UV-inactivated ECTV was confirmed by the inability of the UV light-exposed viruses to produce a cytopathic effect on the monolayers of Vero cells.

### Plasmids

The murine cGas gene was amplified and cloned into the pCMV-Tag2b vector with a FLAG tag on the N terminus. Expression plasmids for HA-tagged Sting-wt (S-wt) (puno1ha-mstingwt) and Sting-gt (puno1-msting-gt) were purchased from the InvivoGen company, and their expressions were confirmed with immunoblotting. The IFN-β reporter and pRL-TK control plasmids for the reporter luciferase assays used in the study were described elsewhere (38). All constructs were confirmed using DNA sequencing.

### Transfection and Luciferase Reporter Assay

Transient transfection was carried out using the FuGENE^®^ HD transfection reagent (E2311, Promega) following the manufacturer’s instructions. HEK293T cells were seeded in 96-well plates at a density of 1 × 10^5^ cells/well and cultured until the cells reached approximately 70–80% confluency. Next, 10 ng of pRL-TK renilla luciferase reporter plasmid and 100 ng of IFN-β firefly luciferase reporter plasmid were transfected together with 100 ng of the indicated expression plasmids. After a 24-h transfection, cells were stimulated with poly(dA:dT)/LyoVec (2 µg/mL), ISD/LyoVec (1 µg/mL), 2′3′-cGAMP (20 µg/mL), and ECTV (MOI of 1) for 15 h, respectively. Luciferase activity was determined using the Dual-Glo^®^ Luciferase Assay System (E2920, Promega).

### RNA Isolation and qRT-PCR

Total RNA was extracted from cellular or tissue (spleen) samples using TRIzol reagent (Invitrogen), and first-strand cDNA was synthesized using the PrimeScript RT reagent kit (RR047A, TaKaRa) following the manufacturer’s instructions. SYBR Green premix (RR820A, TaKaRa) was used for qRT-PCR with a Two Step Real-Time PCR Detection System (Bio-Rad). The primer sequences used for the qPCR were as follows: *Ifn-*α*4*, 5′-CCTGTGTGATGCAGGAACC-3′ and 5′-TCACCTCCCAGGCACAGA-3′; *Ifn-*β, 5′-CAGCTCCAAGAAAGGACGAAC-3′ and 5′-GGCAGTGTAACTCTTCTGCAT-3′; *Ifit1*, 5′-ACAGCAACCATGGGAGAGAATGCTG-3′ and 5′-ACGTAGGCCAGGAGGTTGTGCAT-3′; β*-actin*, 5′-GGCTGTATTCCCCTCCATCG-3′ and 5′-CCAGTTGGTAACAATGCCATGT-3′. Data were normalized to the mRNA levels of the housekeeping gene β-actin. Relative gene expression data were analyzed using the 2^−ΔΔCt^ method.

### RNA Interference

Chemically synthesized siRNA duplexes were obtained from Gene-Pharma and transfected using the FuGENE^®^ HD transfection reagent (E2311, Promega) according to the manufacturer’s instructions. Briefly, a total of 1.0 × 10^5^ L929 or NIH3T3 cells were seeded onto 12-well plates and transfected at a density of 80% with a final concentration of 100 nM of the indicated siRNAs or si-NC. The siRNA oligonucleotides were as follows: si-Sting, 5′-CGAAAUAACUGCCGCCUCATT-3′; si-cGas1, 5′-GAUUGAGCUACAAGAAUAUTT-3′; si-cGas2, 5′-GAGGAAAUCCGCUGAGUCATT-3′; and si-NC (negative control), 5′-UUCUUCGAACGUGUCACGUTT-3′.

### Enzyme-Linked Immunosorbent Assay (ELISA)

RAW264.7 cells and peritoneal macrophages were stimulated with virus or other reagents for the indicated times. Cell culture supernatants and mouse serum were collected, and levels of mouse IFN-β (439407, BioLegend) were measured according to manufacturer’s instructions.

### Western Blot Analysis

For the preparation of soluble cell extracts, harvested cells were washed two times with cold phosphate-buffered saline (PBS) and then were lysed in RIPA buffer containing protease and phosphatase inhibitors (P0013, Beyotime Biotechnology, China). The protein concentration of cell lysates was determined by bicinchoninic acid (BCA) assay (QuantiPro BCA Assay Kit, Sigma-Aldrich) according to the manufacturer’s instructions. Equal amount of total protein (25 µg) was resolved by electrophoresis on 12% Bis–Tris polyacrylamide gels (Shanghai Sangon Biotech, China) and transferred to polyvinylidene fluoride membranes (Immobilon-P Transfer membranes, Millipore). Membranes were blocked for 2 h at room temperature in 5% (wt/vol) Tris-buffered saline supplemented with 0.1% Tween 20 (TBST)-diluted bovine serum albumin (BSA, Amresco) buffer. Membranes were incubated with primary antibody diluted in 5% (wt/vol) BSA and 1× TSBT at 4°C overnight. The primary antibodies used include anti-HA (66006-1, Proteintech), anti-FLAG (F3165, Sigma-Aldrich), anti-β-actin (60008-1, Proteintech), anti-cGas (#31659, Cell Signaling Technology), anti-Irf3 (#4302, Cell Signaling Technology), anti-Sting (#13647, Cell Signaling Technology), anti-Tbk1 (#3504, Cell Signaling Technology), anti-phospho-Irf3 (#4947, Cell Signaling Technology), and anti-phospho-Tbk1 (#5483, Cell Signaling Technology). Antibody signals were detected by the enhanced chemiluminescence detection kit (#1705062, Bio-Rad) after incubation with an appropriate secondary antibody conjugated to horseradish peroxidase. All the membranes were imaged using the ChemiDoc XRS^+^ system (Bio-Rad).

### Mice and Animal Experiments

Specific pathogen-free C57BL/6 mice (B6 strain) between 6 and 10 weeks of age were purchased from the Laboratory Animal Center of Lanzhou Veterinary Research Institute (LVRI), Chinese Academy of Agriculture Science (CAAS). The *cGas^−/−^* [B6(C)-*Mb21d1^*tm1d(EUCOMM)Hmgu*^*/J], *Sting^gt/gt^* (C57BL/6J-*Tmem173^gt^*/J), *Irf3^−/−^* (B6; 129S6-*Irf3^tm1Ttg^*/TtgRbrc), and *Tlr9^−/−^* (C57BL/6J-*Tlr9^M7Btlr^*/Mmjax) mice were originally purchased from the Jackson Laboratory and were bred at the Laboratory Animal Centre of LVRI, CAAS. Mice aged between 6 and 10 weeks were challenged with 3 × 10^3^ or 1.0 × 10^6^ plaque-forming units (PFU) of virus per mouse by subcutaneous injection into the left footpad. Serum of infected mice was collected at 6, 12, 24, and 48 h post-infection (hpi) and 3, 5, and 7 days post-infection (dpi) for ELISA assay. For the determination of survival, the mice were checked daily. For viral titer, a portion of the spleen or liver was removed aseptically from the mice and frozen at −70°C. The tissues from each mouse were weighted and homogenized in PBS to a 10% (wt/vol) lysate. The lysate was frozen and thawed three times and titrated by the plaque-forming assay.

### Generation of Murine Peritoneal Macrophages

Murine peritoneal macrophages were generated as described (39). Briefly, C57BL/6 mice (WT), *Tlr9^−/−^, cGas^−/−^, Sting^gt/gt^*, and *Irf3^−/−^* mice were injected with 1 mL of 3.8% Brewer thioglycollate medium (T9032, Sigma-Aldrich) into the peritoneal cavity for 5 days. Then mice were euthanized by cervical dislocation and pull back the abdominal skin to expose the transparent peritoneal skin. Macrophages were collected by using syringes to inject cold DPBS into the peritoneal cavity of each mouse. The peritoneal fluid was centrifuged for 10 min to remove the supernatant. Cells were resuspended and cultured in 12-well plates at a density of 1 × 10^6^ cells/well. After an 18-h incubation, cells were infected with ECTV (MOI, 5) for 15 or 18 h. Subsequently, the culture media were collected for ELISA, and cells were harvested for western blot analysis.

### ECTV Genomic DNA Copy Number and Viral Titer Measurements

Genomic DNA was extracted from the mouse spleen or liver tissues using a viral RNA/DNA extraction kit (9766, TaKaRa) according to manufacturer’s protocol. ECTV genomic DNA copy numbers were determined by qPCR using ECTV P4b gene specific primers: 5′-GTAGAACGACGCCAGAATAAGATA-3′ and 5′-AGAAGATATCAGACGATCCACAATC-3′. A standard curve was established from a cloned DNA fragment of the ECTV P4b gene (40). Cycle threshold (Ct) values obtained by the real-time PCR were plotted on the standard curve to calculate the viral DNA copy number. The titer of ECTV in the cellular and tissue samples was measured by the plaque assay using Vero cells in 12-well plates. Several 10-fold serial dilutions of the samples were added to individual wells of Vero cell monolayers for 2 h. After adsorption, the supernatants were removed, washed three times in PBS, and then the cells were incubated with 1.0% (wt/vol) high-viscosity carboxy-methyl cellulose (Sigma-Aldrich). At 6 dpi, cells were fixed with 4% formalin for 4 h and then stained with 0.5% crystal violet solution for 20 min to visualize plaques.

### Histological Analysis

Livers were harvested and fixed with 10% neutral buffered formalin solution and then were embedded in paraffin. The paraffin-embedded specimens were cut into 5-µm sections and then stained with hematoxylin and eosin. Each slide with the samples was photographed with a digital optical microscope (Olympus, Tokyo, Japan).

### Statistical Analysis

Data are expressed as means ± SD. Statistical analyses were performed by one-way analysis of variance followed by the Duncan’s multiple range test using the SPSS software (SPSS 18.0 for Windows; SPSS, Chicago, IL, USA). For survival experiments, we used the log-rank (Mantel–Cox). In all figures, ND, not detected; ns, not significant; *P ≤ 0.05; **P ≤ 0.01; and ***P ≤ 0.001.

## Results

### ECTV-Induced Type I IFN Production in L929 and RAW 264.7 Cells, but Not in NIH3T3 Cells

To verify that an ECTV infection can induce type I IFNs production, three cell lines including NIH3T3, L929, and RAW 264.7 were chosen for determining the expression of type I IFNs during the ECTV infection. We found that ISD or ECTV-induced IFN-β production in L929 and RAW264.7 cells, but not in NIH3T3 cells (Figure S1A in Supplementary Material). To assess the time course of the induction of type I IFNs expression by the ECTV infection, RAW264.7 cells were infected with ECTV at an MOI of 5 and harvested for measuring the levels of IFN-α4 and IFN-β transcript using qRT-PCR at the indicated time points. The expression dynamics of *IFN-*α*4* and *IFN-*β showed that the mRNA levels of the two cytokines were upregulated and peaked at 18 hpi (Figures S1D,E in Supplementary Material), which was consistent with the protein levels of IFN-β as determined by ELISA (Figure S1F in Supplementary Material). In addition, a dose dependency of type I IFNs induced by ECTV in RAW264.7 cells was also confirmed using increasing doses of virus from an MOI of 0.1–5. As shown in Figures S1B,C in Supplementary Material, the expression levels of IFN-α4 and IFN-β were increased with higher doses of ECTV, which reached the highest level at an MOI of 5. We then used an MOI of 5 for the ECTV infection in the rest of the *in vitro* infection experiments reported in this paper.

### Sting and cGas Are Required for the Induction of IFN-β During the ECTV Infection

**Figure 1:**
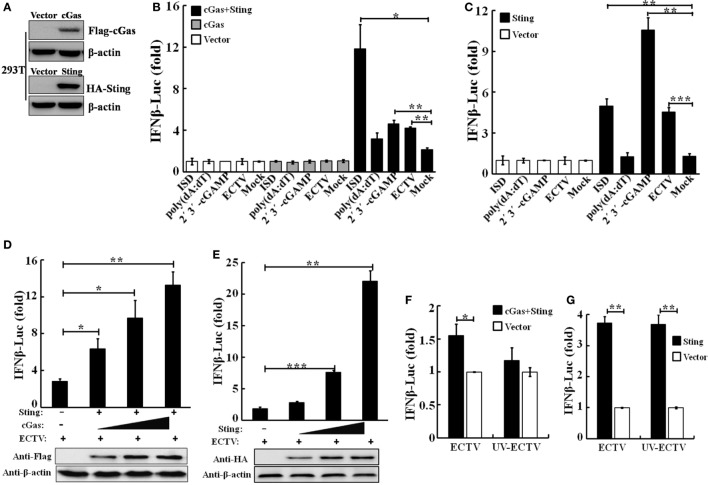
Sting and cGas are required for the induction of IFN-β during ectromelia virus (ECTV) infection. **(A)** Overexpressed cGas, Sting, and β-actin protein levels in HEK293T cells were evaluated by Western blot. HEK293T cells (1 × 10^5^) were seeded in a 96-well plate and then were transfected with pRL-TK *Renilla* luciferase reporter plasmid (10 ng/well), IFN-β firefly luciferase reporter plasmids (100 ng/well), and together with cGas (100 ng/well), Sting (100 ng/well), cGas + Sting (50 ng per plasmid per well), or vector only (100 ng/well) **(B,C)**. After a 24-h transfection, cells were either stimulated with poly(dA:dT)/LyoVec (2 μg/mL), ISD/LyoVec (1 μg/mL), 2′3′-cGMP-AMP (cGAMP) (20 μg/mL), or infected with ECTV (MOI of 1) for 15 h, respectively. Luciferase activity was determined using the Dual-Glo^®^ Luciferase Assay System. **(D)** HEK293T cells were transfected with Sting expressing plasmid (50 ng/well), and increasing doses of plasmids expressing cGas (25, 50, and 100 ng/well) combined with decreasing doses of vector (75, 50, and 0 ng/well). **(E)** HEK293T cells were transfected with increasing doses of plasmids expressing Sting (25, 50, and 100 ng/well) combined with decreasing doses of vector (75, 50, and 0 ng/well). After a 24-h transfection, cells were infected with ECTV at an MOI of 1 for 15 h. Expression of transfected cells with increasing doses cGas or Sting was examined by immunoblot analysis. HEK293T cells were transfected with cyclic GMP-AMP synthase (cGAS) (50 ng) combined with Sting (50 ng) expressing plasmids **(F)** or Sting (100 ng) expressing plasmids **(G)**, and 24 h later, cells were infected with ECTV and UV-inactivated ECTV at an MOI of 1 for 15 h. Luciferase activity was determined using the Dual-Glo Luciferase Assay System. All the data were averaged from three independent experiments in biological triplicate and represents mean ± SD. Statistical analyses were performed by a *t*-test **(F,G)** or one-way analysis of variance followed by the Duncan’s multiple range test (*P ≤ 0.05; **P ≤ 0.01; and ***P ≤ 0.001).

In view of the importance of the cGAS–STING pathway in the production of type I IFNs upon infections by DNA virus, we hypothesize that the type I IFN gene expression induced by ECTV might be through cGas and Sting. To investigate the roles of murine cGas and Sting in response to the ECTV infection, we first compared the ability of ECTV to stimulate cGas-induced pathways in human cells. HEK293T cells were transfected with murine cGas or in combination with Sting, and then infected/stimulated with ECTV, poly(dA:dT), ISD, or 2′3′-cGAMP. The protein levels of cGas and Sting overexpressed in HEK293T cells were confirmed by immunoblotting (Figure 1A). As previously mentioned, cGAS and STING are poorly expressed in HEK293T cells (7, 41). As predicted, the increased IFN-β promoter activity was only observed in cGas and Sting co-transfected cells (Figure 1B). Moreover, the induction of the IFN-β promoter showed that all of the three reagents and ECTV have stimulatory potency, and the triggered IFN-β response was entirely Sting-dependent. Consistent with previous studies (42, 43), the magnitude of the IFN-β induction by ISD was higher than others in cGas and Sting co-transfected cells. HEK293T cells were then used to examine the role of Sting in IFN-β expression during the infection. As compared with the co-transfected treatment, HEK293T cells transfected with Sting alone induced at lower levels of IFN-β expression (Figure 1C), suggesting the Sting expression alone cannot entirely drive IFN-β expression.

**Figure 2:**
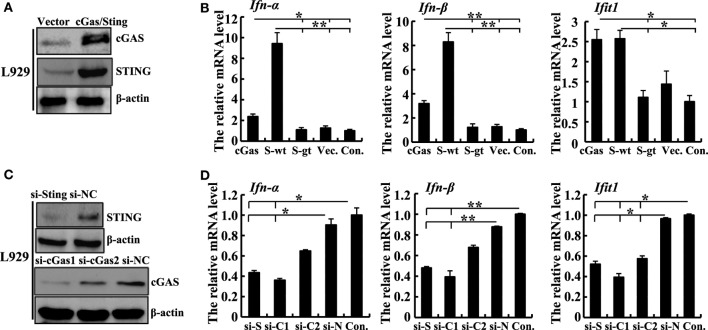
Sting and cGas are required for the induction of IFN-β during ectromelia virus (ECTV) infection in L929 cells. **(A)** Western blot analysis of overexpressed cGas, Sting, and β-actin protein levels in L929 cells. **(B)** L929 (2 × 10^5^) cells were seeded in a 12-well plate and then were transfected with cGas, Sting-wt (S-wt), Sting-gt (S-gt), or empty (Vec.) plasmids. Thirty hours after transfection, cells were infected with ECTV (MOI of 5) for 18 h, and then the mRNA levels of IFN-α, IFN-β, and Ifit1 were analyzed by qPCR. **(C)** Western blot analysis of siRNA knockdown of cGas, Sting, and β-actin protein levels in L929 cells. **(D)** L929 (2 × 10^5^) cells were seeded in a 12-well plate and then were transfected with siRNAs for cGas (si-C1 and si-C2), Sting (si-S), or si-NC (si-N). Thirty-six hours after transfection, cells were infected with ECTV (MOI of 5) for 18 h, and then the mRNA levels of IFN-α, IFN-β, and Ifit1 were analyzed by qPCR. All the data represent mean ± SD of biological triplicates from at least three independent experiments. Statistical analyses were performed by one-way analysis of variance followed by the Duncan’s multiple range test. Con. means control group, which cells were only infected with ECTV (MOI of 5). In this figure, *P ≤ 0.05 and **P ≤ 0.01.

To validate the function of the cGas–Sting pathway in the activation of IFN-β with ECTV, the induction of IFN-β expression by cGas and Sting in a dose-dependent manner was observed during the ECTV infection (Figures 1D,E). In addition, we tested the induction of the IFN-β promoter activated by ECTV or UV-inactivated ECTV in the presence of cGas (50 ng) and/or Sting (50 or 100 ng) (Figures 1F,G). As shown in Figures 1F,G, UV-inactivated ECTV can also induce the expression of IFN-β. As the host range of ECTV is restricted, we next investigated the roles of cGas and Sting in response to ECTV infection in murine cells. NIH3T3 and L929 cells were transfected with expression plasmids or siRNAs for cGas or Sting, and then were infected with ECTV. Overexpression or knockdown of cGas and Sting in these two cell lines were confirmed by immunoblotting as shown in Figure 2 and Figure S2 in Supplementary Material. Overexpression of cGas or S-wt resulted in the increased induction of IFN-α, IFN-β, and Ifit1 mRNA levels in ECTV-infected L929 cells, but which were non-existent in cells transfected with Sting-gt (a point mutation that results in the loss of Sting expression) (Figure 2B). Expectedly, knockdown of either cGas or Sting decreased the induction of IFN-α, IFN-β, and Ifit1 mRNA levels in L929 cells with the ECTV infection (Figure 2D). However, the mRNA levels of the three molecules (IFN-α, IFN-β, and Ifit1) showed no significant changes in NIH3T3 cells, and thus may not have a function in the cGas–Sting pathway in this cell line (Figures S2B,D in Supplementary Material). Therefore, this suggests that the cGAS and STING may be responsible for sensing ECTV infections and inducing type I IFN expression.

### ECTV Infection Induces the Phosphorylation of Tbk1 and Irf3

**Figure 3:**
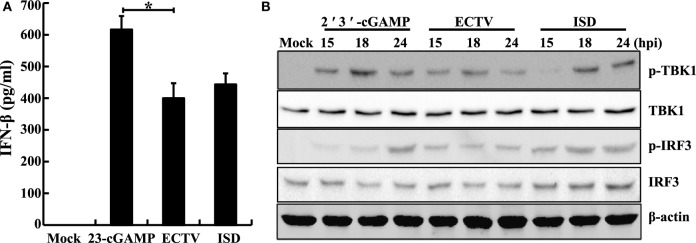
Ectromelia virus (ECTV) infection induces the phosphorylation of Tbk1 and Irf3. RAW264.7 cells (1 × 10^6^) were either stimulated with poly(dA:dT)/LyoVec (2 μg/mL), ISD/LyoVec (1 μg/mL), 2′3′-cGMP-AMP (cGAMP) (20 μg/mL), or infected with ECTV (MOI of 5). The supernatants were collected at 18 h post-infection (hpi) for determining the concentrations of IFN-β by using enzyme-linked immunosorbent assay (ELISA) **(A)**, and cells were collected at 15, 18, and 24 hpi. All the collected cells were used for western blot analysis using anti-phospho-Tbk1, anti-Tbk1, anti-phospho-Irf3, and anti-Irf3 **(B)**. β-Actin was used as a loading control. The ELISA data were averaged from three independent experiments in biological triplicate. The data were analyzed using a one-way analysis of variance followed by the Duncan’s multiple range test. Results of western blot analysis shown are representative of three independent experiments (*P ≤ 0.05).

It is well known that TBK1 and IRF3 are two important factors of multiple antiviral signaling pathways, including cytosolic DNA sensor signaling such as through the cGAS–STING signaling pathway (44). We performed western blot analysis of ECTV-infected L929, NIH3T3, and RAW264.7 cells, and found that the ECTV infection triggered Tbk1 and Irf3 phosphorylation in RAW264.7 and L929 cells, which reached peaks at 18 hpi (Figures S3A,B in Supplementary Material), whereas it failed to do so in NIH3T3 cells (Figure S3C in Supplementary Material), indicating that ECTV induced the levels of phosphorylation of Tbk1 and Irf3 in L929 and RAW264.7 cells in a cell type-dependent manner (15, 45). In addition, upon stimulation with ISD and 2′3′-cGAMP, the Irf3 and Tbk1 were phosphorylated, and IFN-β was secreted in RAW264.7 cells (Figures 3A,B). Notably, much higher levels of phosphorylation of Tbk1 and Irf3 were stimulated by ISD and 2′3′-cGAMP than by the ECTV infection, indicating that ECTV might express some potent immunomodulators that are involved in the modulation of TBK1 and IRF3 phosphorylation. Alternatively, this may be due to a stronger stimulation with the concentrations of cGAMP or ISD.

### The cGas–Sting–Tbk1–Irf3 Pathway Drives the Early IFN Response to ECTV

**Figure 4:**
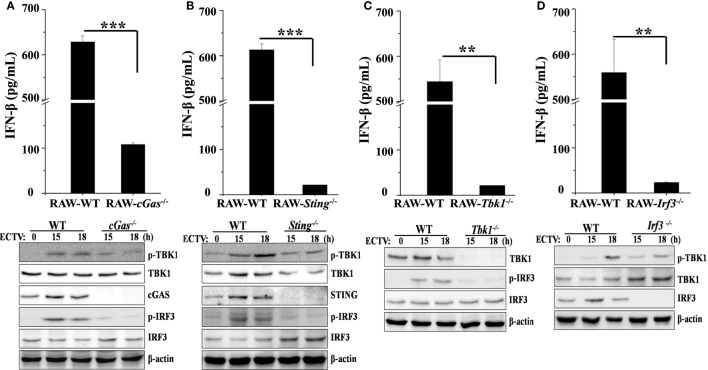
The cGas–Sting–Tbk1–Irf3 pathway contributes to the induction of IFN-β in RAW264.7 cells by ectromelia virus (ECTV). *cGas^−/−^* **(A)**, *Sting^−/−^* **(B)**, *Irf3^−/−^* **(C)**, and *Tbk1^−/−^* **(D)** RAW264.7 and wild-type (WT) cells were infected with ECTV at an MOI of 5. Supernatants were collected 18 h post-infection (hpi) for determining the concentrations of IFN-β by using enzyme-linked immunosorbent assay (ELISA), and cells were collected at 15 and 18 hpi for western blot analysis using anti-phospho-Tbk1, anti-Tbk1, anti-phospho-Irf3, and anti-Irf3. β-Actin was used as a loading control. The ELISA data are averaged from three independent experiments in biological triplicate. Results of western blot analysis shown are representative of three independent experiments. The data were analyzed using a *t*-test on SPSS software (**P ≤ 0.01 and ***P ≤ 0.001).

The critical role of Tlr9 for type I IFNs induction in ECTV-infected mice has been recently emphasized (32). Other PRRs, such as DAI, a receptor upstream of Sting, has been ruled out for driving the expression of type I IFNs during ECTV infections (32). As previously described, cGAS acts as a general cytosolic DNA sensor upstream of STING that is responsible for the recognition of several DNA viruses, including VACV, modified vaccinia virus Ankara (MVA), herpes simplex virus 1 (HSV-1), murine gamma-herpesvirus 68 (MHV68), Kaposi’s sarcoma-associated herpesvirus, adenovirus, human papilloma viruses, hepatitis B virus, and human cytomegalovirus (36, 41, 46–50). However, whether the cGas also recognizes ECTV and induces the production of type I IFNs are largely unknown so far. Subsequently, to investigate the contributions of the cGas–Sting–Tbk1–Irf3 pathway for driving the production of type I IFNs during the ECTV infection, *cGas^−/−^, Sting^−/−^, Tbk1^−/−^*, and *Irf3^−/−^* RAW264.7 cells were challenged with ECTV, and the secretion of IFN-β and levels of phosphorylation of IRF3 and TBK1 were detected by ELISA and immunoblotting, respectively. As shown in Figures 4A,B, we found that ECTV-induced IFN-β secretion was reduced by nearly 82% in *cGas^−/−^* cells, while the *Sting^−/−^* cells nearly abolished the secretion of IFN-β, which suggests the *cGas* deficiency might be partially complemented by other receptors upstream of STING, such as IFI16 (mouse: Ifi204) and DDX41. Similar to *Sting^−/−^* cells, ECTV-induced IFN-β secretion was barely detectable in *Tbk1^−/−^* and *Irf3^−/−^* cells (Figures 4C,D, respectively). In addition, the phosphorylation levels of IRF3 and TBK1 were not detected in these knockout cells by western blot analysis. As compared with WT cells, Tbk1 and Irf3 phosphorylation were severely impaired in cGas-deficient cells (Figure 4A). Consistently, the phosphorylation of Irf3 and Tbk1 in *Sting^−/−^* cells appeared weaker than in the WT cells (Figure 4B). As expected, cells deficient in Tbk1 or Irf3 appeared to have weaker levels of Tbk1 or Irf3 phosphorylation, respectively (Figures 4C,D), indicating other pathways involved in Tbk1 or Irf3 were activated during the ECTV infection.

**Figure 5:**
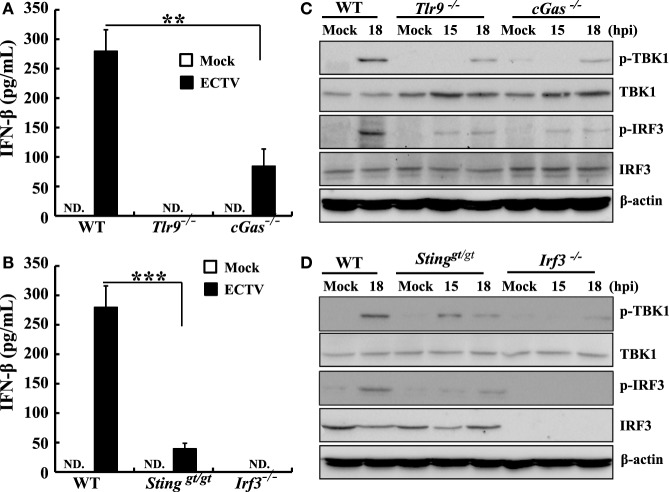
Murine peritoneal macrophages response ectromelia virus (ECTV) infection was dependent on Tlr9 and cGas–Sting–Irf3 pathway. Peritoneal macrophages generated from wild-type (WT), *cGas^−/−^, Sting^gt/gt^, Tlr9^−/−^*, and *Irf3^−/−^* mice were non-infected or infected with ECTV at an MOI of 5. Supernatants were collected 18 h post-infection (hpi) for determining the concentrations of IFN-β by using enzyme-linked immunosorbent assay (ELISA) **(A**,**B)**, and cells were collected at 15 and 18 hpi for western blot analysis using anti-phospho-Tbk1, anti-Tbk1, anti-phospho-Irf3, and anti-Irf3 **(C**,**D)**. β-Actin was used as a loading control. The ELISA data were averaged from two independent experiments in biological triplicate. Results of western blot analysis shown are representative of two independent experiments. The data were analyzed using a *t*-test on SPSS software. In this figure, ND, not detected; **P ≤ 0.01 and ***P ≤ 0.001.

Furthermore, to confirm these results in primary cells, peritoneal macrophages were generated from WT, *Tlr9^−/−^, cGas^−/−^, Sting^gt/gt^*, and *Irf3^−/−^* mice and the type I IFN induction and phosphorylation of TBK1 or IRF3 upon ECTV infection were evaluated. As shown in Figures 5A,B, the IFN-β secretion was only detected in WT, *cGas^−/−^*, and *Sting^gt/gt^* cells. As compared with WT cells, ECTV-induced IFN-β secretion was reduced by 70 and 86% in *cGas^−/−^*, and *Sting^gt/gt^* cells, respectively, while the *Tlr9^−/−^* and *Irf3^−/−^* cells abolished the secretion of IFN-β. Similarly, the phosphorylation of TBK1 or IRF3 induced by ECTV were severely impaired in all these gene knockout peritoneal macrophages (Figures 5C,D). Taken together, these results definitively show that ECTV infection is capable of activating the cGas–Sting–Tbk1–Irf3 axis and Tlr9, leading to the transcription of type I IFNs.

### Sting and cGas Restrict ECTV Replication in RAW264.7 Cells

**Figure 6:**
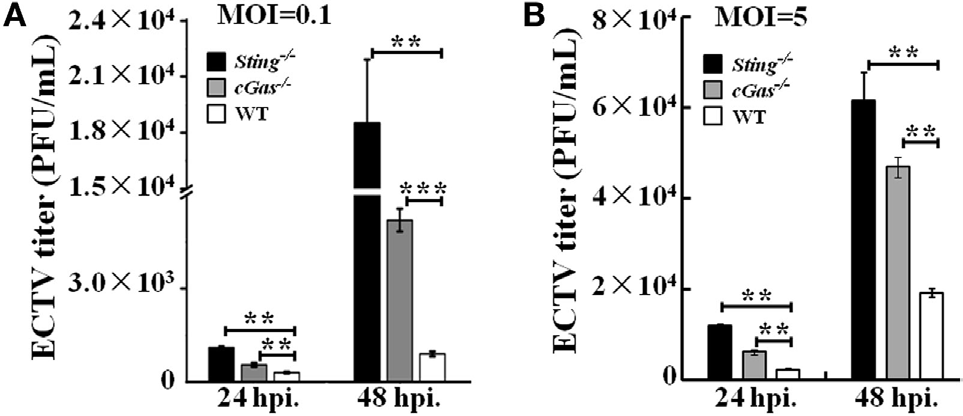
Sting and cGas restrict ectromelia virus (ECTV) replication in RAW264.7 cells. *Sting^−/−^, cGas^−/−^*, and wild-type (WT) cells were infected with ECTV at an MOI of 0.1 **(A)** or 5 **(B)**. After a 2-h incubation, the supernatants were replaced with fresh medium supplemented with 1% fetal bovine serum, and then the supernatants were collected at the indicated time points for viral titer measurements by using the plaque assay. All the data represent mean ± SD of biological triplicates from at least three independent experiments. The data were analyzed using a one-way analysis of variance followed by the Duncan’s multiple range test (**P ≤ 0.01 and ***P ≤ 0.001).

It has been previously reported that STING has the ability to control viral replication (15, 51, 52). To address whether the cGas–Sting pathway has the ability to restrict ECTV replication, *cGas^−/−^, Sting^−/−^*, and WT RAW264.7 cells were infected with two doses (MOI of 0.1 and 5) of virus, and viral titers were assessed after 24 and 48 hpi. Significant increases in the amount of viral progeny released were observed in *cGas^−/−^* and *Sting^−/−^* cells compared with WT cells at all time points. At a low MOI of 0.1, cells deficient in Sting showed more increased viral replication than those lacking cGas, which showed a fourfold and twofold increase in *Sting^−/−^* and *cGas^−/−^* cells, respectively, as compared with WT cells at 24 hpi (Figure 6A). At 48 hpi, similar results were observed but a higher enhanced replication was observed in knockout cells (20-fold and 5.7-fold in *Sting^−/−^* and *cGas^−/−^* cells, respectively) (Figure 6A). Moreover, ECTV growth curves were generated at an infection dose of MOI of 5. As compared with a low infection dose, we observed reduced progeny release (3.2-fold and 2.5-fold in *Sting^−/−^* and *cGas^−/−^* cells, respectively) at 48 hpi (Figure 6B). Collectively, these results demonstrate that cGas and Sting have abilities to restrict ECTV replication *in vitro*.

### Sting and cGas Are Critical for Mousepox Resistance *In Vivo*

**Figure 7:**
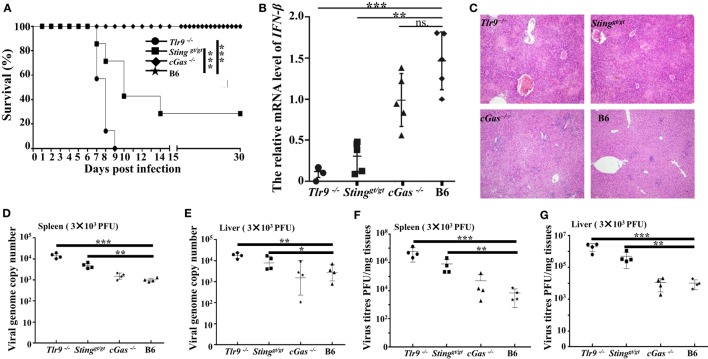
Tlr9 and Sting are critical for resistance to ectromelia virus (ECTV) at a low infection dose. *Sting^gt/gt^, cGas^−/−^, Tlr9^−/−^*, and wild-type (WT) mice (B6) were infected with 3 × 10^3^ plaque-forming units (PFUs) of ECTV in the left footpad. **(A)** Mice were monitored twice daily over 30 days for survival. **(B)** The expression of IFN-β in the spleens of the indicated mice at 3 days post-infection (dpi) was examined by qPCR. **(C)** Liver sections of the indicated mice at 7 dpi were stained with hematoxylin and eosin. **(D–G)** The spleen and liver from infected mice were harvested at 7 dpi, and then the viral genome copy numbers **(D**,**E)** and viral loads **(F**,**G)** were determined by qPCR and plaque assay, respectively. Statistical analyses were performed by one-way analysis of variance followed by the Duncan’s multiple range test. For survival experiments, we used the log-rank (Mantel–Cox). In this figure, ns, not significant; *P ≤ 0.05; **P ≤ 0.01; and ***P ≤ 0.001.

Our *in vitro* studies reveal the cGas-Sting pathway plays an essential role in controlling ECTV infection *via* inducing the production of type I IFNs. To further investigate whether the cGAS–STING axis was required for resistance to mousepox *in vivo, Tlr9^−/−^, cGas^−/−^, Sting^gt/gt^*, and WT C57BL/6 mice were infected with a low (3 × 10^3^ PFU per mouse) and high dose (1.0 × 10^6^ PFU per mouse) of ECTV in the footpad, respectively. We found that after infection with a low dose (3 × 10^3^ PFU per mouse) of ECTV, mice deficient in Tlr9 (*Tlr9^−/−^*) or Sting (*Sting^gt/gt^*) were highly susceptible to mousepox. In agreement with previous studies, death occurred in 100% of *Tlr9^−/−^* but only in 72.4% of *Sting^gt/gt^* mice (Figure 7A). However, mice lacking *cGas* (*cGas^−/−^*) showed no lethality, similar to WT mice. Consistently, viral genome copy number and viral loads in the livers and spleens of *Tlr9^−/−^* and *Sting^gt/gt^* mice but not of *cGas^−/−^* mice were significantly higher than in WT mice (Figures 7D–G). Moreover, the livers of *Tlr9^−/−^* and *Sting^gt/gt^* mice showed severe pathology as determined by histology, which were mild in both *cGas^−/−^* and WT mice (Figure 7C). More infiltration of inflammatory cells and massive necrosis were observed in the livers of *Tlr9^−/−^* and *Sting^gt/gt^* mice. We next assayed the mRNA levels of IFN-β in the spleen at 3 dpi as well as the secretion of IFN-β in the serum of infected mice at 6, 12, 24, and 48 hpi and 3, 5, and 7 dpi. Unfortunately, none of the serum samples from all the infected mice were detectable. The expression of IFN-β in the spleens was significantly lower in *Tlr9^−/−^* and *Sting^gt/gt^* but not in *cGas^−/−^* and WT mice (Figure 7B). Nevertheless, *Tlr9^−/−^* mice expressed significantly lower levels of IFN-β than *Sting^gt/gt^* mice (Figure 7B). Thus, *Tlr9^−/−^* and *Sting^gt/gt^* mice are both critical for resistance to ECTV infection at a relatively low dose.

**Figure 8:**
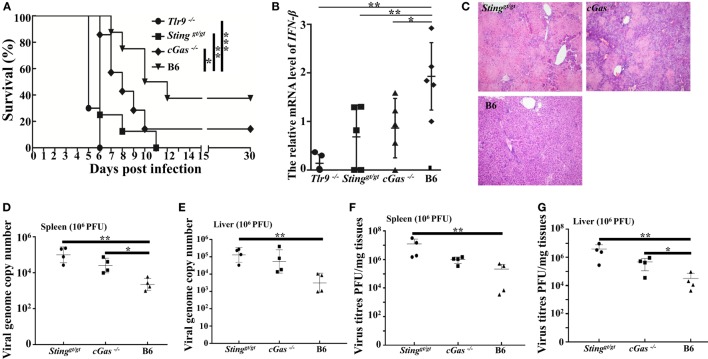
Tlr9, Sting, and cGas are critical for resistance to ectromelia virus (ECTV) at a high infection dose. *Sting^gt/gt^, cGas^−/−^, Tlr9^−/−^*, and wild-type (WT) mice (B6) were infected with 1 × 10^6^ plaque-forming units (PFUs) of ECTV in the left footpad. **(A)** Mice were monitored twice daily over 30 d for survival. **(B)** Expression of IFN-β in the spleens of the indicated mice at 3 days post-infection (dpi) was examined by qPCR. **(C)** Liver sections of the indicated mice at 7 dpi were stained with hematoxylin and eosin. **(D–G)** The spleen and liver from infected mice were harvested at 7 dpi, and then viral genome copy numbers **(D**,**E)** and viral loads **(F**,**G)** were determined by qPCR and plaque assay, respectively. Statistical analyses were performed by one-way analysis of variance followed by the Duncan’s multiple range test. For survival experiments, we used the log-rank (Mantel–Cox). In this figure, ns, not significant; *P ≤ 0.05; **P ≤ 0.01; and ***P ≤ 0.001.

Considering the results obtained from a low dose of infection, we found slightly reduced expression of IFN-β, mild pathology, and higher viral loads in *cGas^−/−^* mice than in WT mice; therefore, a lethal dose of virus (1.0 × 10^6^ PFU per mouse) was used for further animal infections. Similar to the low infection dose, *Tlr9^−/−^* and *Sting^gt/gt^* mice were more susceptible to ECTV infection, but both resulted in 100% mortality. The *cGas^−/−^* mice had a lower survival rate (14%) and succumbed to disease more rapidly than WT mice. All *Tlr9^−/−^* mice died between 5 and 6 dpi, and viral loads, viral genome copy number, and pathology were therefore only performed in the remaining three groups (Figure 8A). Accordingly, vial genome copy number and viral loads in the livers and spleens of *Sting^gt/gt^* and *cGas^−/−^* mice were higher than in WT mice (Figures 8D–G). Meanwhile, the *Sting^gt/gt^* and *cGas^−/−^* mice showed more infiltration of inflammatory cells and more severe bridging necrosis than WT mice as determined by histology (Figure 8C). Furthermore, the expressions of IFN-β in the spleens of *Tlr9^−/−^, Sting^gt/gt^*, and *cGas^−/−^* mice were significantly lower than that in WT mice (Figure 8B). Thus, in addition to Tlr9, the cGas–Sting pathway is also essential for survival against ECTV infections.

## Discussion

Recognition of pathogen-derived DNA is a vital strategy by which the innate immune system responds to microbial invasions. Atypical or mislocalized DNA can engage multiple innate immune pathways to trigger the production of type I IFNs and the establishment of the cellular antiviral state (53). TLR9 is a receptor for unmethylated CpG DNA motifs presents in the endosomal membranes of phagocytic cells. Signaling through Tlr9 is induced by ECTV to produce type I IFNs responses in plasmacytoid dendritic cells (pDCs) but not in classical dendritic cells (cDCs) (33). The recognition of ECTV in a Tlr9-dependent manner to produce type I IFNs has been well established *in vitro* and *in vivo* (32–34). Experimentally, Tlr9 has been concluded to be the only TLR required for mousepox resistance by ruling out other TLR members (32). Recently, Sting, a critical mediator of the cytosolic DNA-sensing pathway, has been confirmed to have essential roles in the resistance to ECTV infections (32). In addition, the cGAS–STING pathway is emerging as the dominant cytosolic DNA-sensing pathway in infections by DNA viruses (5, 8, 19). Furthermore, it has recently been demonstrated that Dai was not the critical DNA sensor upstream of Sting for the recognition of ECTV infection and the induction of type I IFNs (32). However, the receptor upstream of Sting that senses ECTV to drive the expression of type I IFNs has not yet been identified. Therefore, we hypothesized that the cGas–Sting pathway contributes to the production of type I IFNs in ECTV-infected cells.

Using the *in vitro* ECTV-infection model, we have demonstrated that ECTV induces type I IFN production in L929 and RAW264.7 cells, but not in NIH3T3 cells. Similarly, ECTV can stimulate the production of IFN-α in pDCs but not in cDCs (33). Consistent with the determination of the phosphorylation of TBK1 and IRF3, ECTV infections triggered the phosphorylation of Tbk1 and Irf3 in L929 and RAW264.7 cells, whereas it fails to do so in NIH3T3 cells. Moreover, overexpression or knockdown levels of cGas and Sting have no significant effect on type I IFN expression levels in ECTV-infected NIH3T3 cells. As described in previous studies, not all cell lines are able to respond to DNA viruses or cytosolic DNA, and the deficiency or poor expression of some key factors involved in the innate immune response results in the failure of type I IFN production (45). Thus, the NIH3T3 cell line is not suitable for the study of innate immune pathways. In addition, the IFN-β luciferase promoter assay revealed that overexpression of cGas and Sting can induce IFN-β promoter activation following an ECTV infection. Accordingly, the cGas or Sting-activating ligands ISD and 2′3′-cGAMP displayed higher stimulatory potency but not poly(dA:dT). As for poly(dA:dT), this ligand has no effect on the induction of type I IFN in the absence or presence of cGas or Sting, and it is possible that the RNA polyIII–RIG-I pathway is involved in a poly(dA:dT) mediated-IFN response (42, 54). ECTV encodes host–response modifiers (HRMs) of both NF-κB and type I IFN pathways (55–58). The mechanism of action of HRMs to inhibit the host immune response is by disrupting receptor–ligand interactions but also acts through impeding cytokine secretion or modulating post-ligation signaling (55, 56). Thus, the lower levels of IFN-β expression induced by ECTV may result from the modification of the type I IFN pathways by ECTV-encoded HRMs. Alternatively, a higher stimulatory potency of ISD and 2′3′-cGAMP is observed because they are “pure” ligands. Moreover, compared with ISD and 2′3′-cGAMP, the lower levels of phosphorylation of Irf3 and Tbk1 induced by ECTV needs to be more fully confirmed in future studies.

The STING has been identified as a pivotal signaling adaptor for the cytosolic DNA sensors and the induction of type I IFN. Besides cGAS, a series of studies have established several other cytosolic DNA sensors utilizing STING as an adaptor, including IFI16 (mouse: Ifi204), DAI, and DDX41 (54, 59, 60). We found nearly 97 and 86% reduced IFN-β induction in ECTV-infected *Sting^−/−^* RAW264.7 cells and *Sting^gt/gt^* peritoneal macrophages, respectively, whereas IFN-β induction was reduced by 82% in cGas-deficient RAW264.7 cells and 70% in *cGas^−/−^* peritoneal macrophages, suggesting other Sting-dependent DNA sensors might also be involved in the IFN-β production. However, Dai has been ruled out as the DNA receptor upstream of Sting for the recognition of ECTV *in vivo* because no differences were observed in the survival rate and type I IFNs expression between *Dai^−/−^* and WT mice (32). We have not yet determined the contributions of Ifi204 and Ddx41 to the induction of type I IFN production in ECTV infections. However, data from modified vaccinia virus Ankara (MVA), an attenuated VACV also belonging to OPV, showed that DDX41 is required for the MVA-induced phosphorylation of TBK1 and IRF3 in macrophages, and no significant differences were observed for MVA-induced type I IFN production in IFI16-deficient cDCs, as compared with WT cells (36). Thus, the exact functions of Ifi204 and Ddx41 or other specific candidate receptors in the resistance to ECTV need to be investigated in the future. Alternatively, an STING-dependent but cGAS-independent pathway was reported in the production of type I IFN and ISGs, which was stimulated by membrane perturbation, including virus–cell fusion, liposome–cell fusion or cell–cell fusion that act as a danger signal (61, 62). Here, the different reduction of IFN-β in Sting-deficient and cGas-deficient macrophages could originate from the capacity of STING to sense the viral particles directly. Therefore, it should be pointed out that an indirect activity of cGas cannot be ruled out for the production of type I IFN through STING-dependent but cGAS-independent pathway in this study.

**Figure 9:**
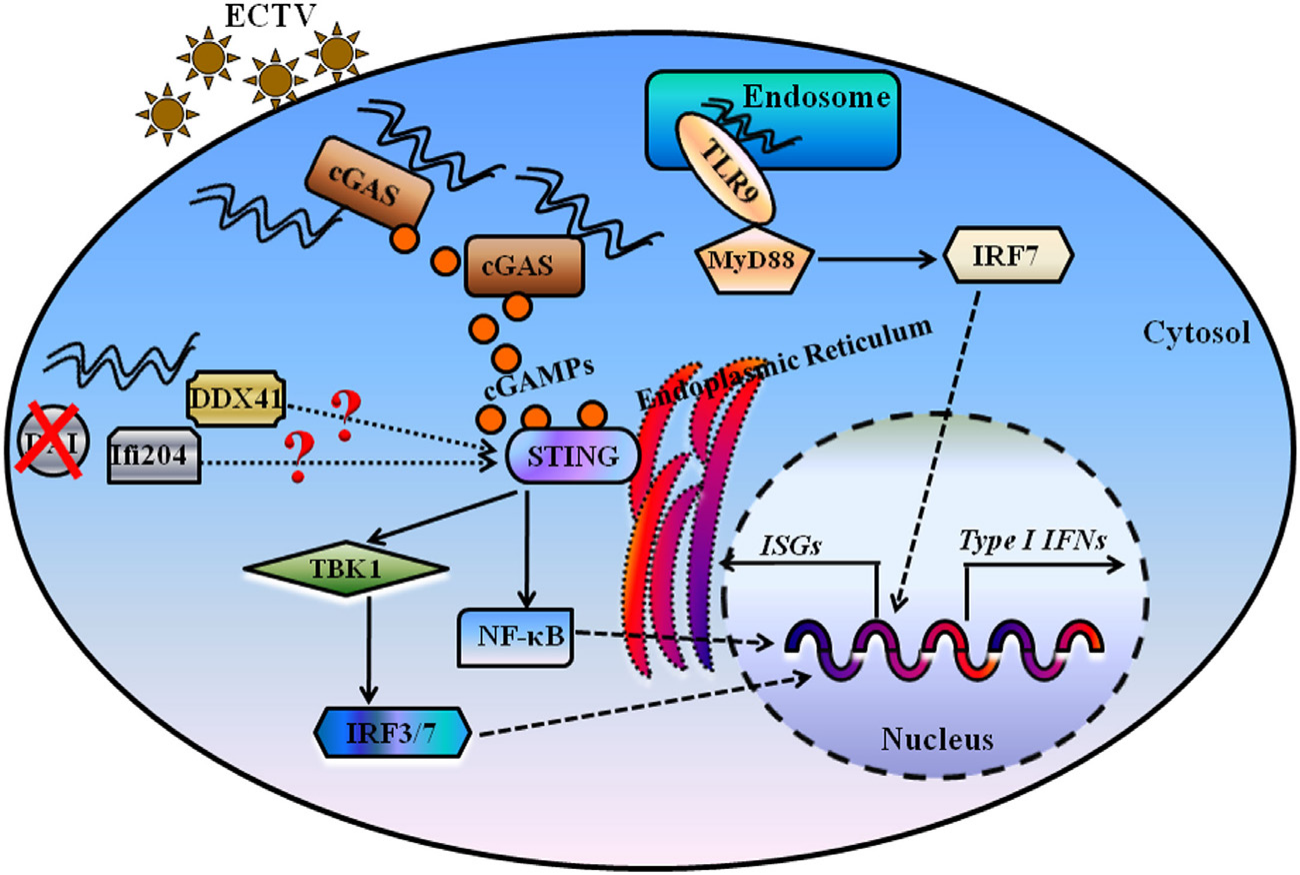
Proposed model for illustrating the essential roles of DNA sensors in recognition and innate interferons (IFNs) production during ectromelia virus (ECTV) infection. Upon viral entry, some of ECTV virions are processed in the endosome, and viral DNA is firstly detected by Tlr9, which signals through the Myd88–Irf7 axis to promote expression of type I IFNs. Moreover, some of viral DNA is detected by the cytosolic DNA sensor cGas, and cGas catalyzes the formation of cGMP-AMP (cGAMP), which through the Sting–Tbk1–Irf3/7 axis leads to the activation of IFN responses. Other cytosolic DNA sensors, including Ifi204 and Ddx41, potentially recognize viral DNA present in the cytoplasm for the activation of IFN responses. DAI, one of the receptors upstream of Sting, has been ruled out as the critical DNA sensor for the activation of IFN responses during an ECTV infection.

ECTV causes a lethal infection to mice but displays different pathogenicity among different mouse genotypes (63). It was established that C57BL/6 mice were classified as resistant, with a 50% lethal dose (LD_50_) > 10^5^ PFU, whereas BALB/c mice are susceptible (LD_50_ < 10 PFU) to ECTV infection (64). Using two infection doses, 3 × 10^3^ PFU and 10^6^ PFU, data from the survival rates, pathology, and IFN-β induction of infected mice revealed the importance of Tlr9 in the resistance to mousepox, which is consistent with the recent finding that Tlr9 is the only TLR required for resistance to mousepox. By contrast, the role of cGas in the resistance to mousepox is not outstanding at the low dose of infection, but its importance was highlighted at the high dose of infection, which suggests that a weak or no role of cGas–Sting pathway is involved at a low dose of infection. However, mice deficient in Sting are more susceptible to mousepox than cGas-deficient mice, suggesting other Sting-dependent pathways might sense ECTV to drive type I IFN expression. As mentioned earlier, Dai has been ruled out, and Ifi204 and Ddx41 need to be further evaluated. Fortunately, it has been established that the production of IFN-α is mostly through Sting-Irf3 and the production of IFN-β is through Sting–NF-κB (31, 32). We therefore postulate that when the viral particle fuses to the cell membrane, the ECTV genomic DNA are endocytosed and are sensed by TLR9 in the endosomal/lysosomal compartment. On the other hand, the ECTV capsid is broken down in the cytoplasm, triggering leakage of the viral DNA into the cytoplasm and leading to the detection of viral DNA by cGas and other cytosolic DNA sensors, and the expression of type I IFNs through the Sting adapter (Figure 9). In addition, it has been shown that TLR9 contributes to the production of type I IFNs to systemic viral infections, while cytosolic nucleic acid sensors including cGAS, IFI16 (mouse: Ifi204), RIG-I, and MDA5 mediate local immune responses to viral infections (36). Notably, the secretion of IFN-β in the serum of all mice through a subcutaneous infection was undetectable in our study, suggesting the cGas–Sting pathway might be responsible for local type I IFNs production at the early stage of ECTV infections. Furthermore, at the late stage of viral infections, Tlr9 may contribute to the production of type I IFNs in a systemic ECTV infection. Moreover, AIM2 is another cytosolic DNA sensor but activates ASC-caspase-1-dependent inflammasome in response to cytosolic dsDNA, leading to the generation of IL-1β and IL-18 (65, 66). However, AIM2 inflammasome can attenuate cGAS–STING-mediated type I IFNs production in resistance to VACV, HSV-1, VSV, and SeV infections (8). Also, we found that *Aim2^−/−^* and *caspase-1^−/−^* mice were resistant to ECTV infection compared with the WT mice, which was associated with lower viral loads and milder pathology in the spleens and livers of *Aim2^−/−^* and *caspase-1^−/−^* mice (unpublished data). Interestingly, a recent published study has reported that Irf7 is necessary for Tlr9–Myd88 and Sting–Irf7/NF-κB pathway the expression of pro-inflammatory cytokines and type I IFNs, respectively, but not Irf3, with ECTV infection *in vivo* (31, 32). However, we found that Irf3 is required for the ECTV-induced type I IFN production *in vitro*, but the roles of Irf3/7 in the induction of pro-inflammatory cytokines and type I IFNs are not investigated *in vitro* and/or *in vivo*, and would need to be confirmed in future studies. In addition, cGAS triggers innate immune responses through the production of the second messenger cGAMP, which binds and activates the STING, and ultimately leads to induction of type I IFNs by the activation of the TBK1 and IRF3. Then, further studies are needed to elucidate whether the cGAMP is produced upon ECTV infection.

In summary, we present data demonstrating the critical role of the cGas–Sting pathway in ECTV-induced type I IFN production *in vitro* and *in vivo*. ECTV was sensed by cGas and induced the production of type I IFN through the Sting–Tbk1–Irf3 axis to restrict the replication of virus. Although the importance of the Tlr9–Myd88 pathway in mousepox resistance has been well established, the cytosolic DNA-sensing pathways, especially the cGas–Sting pathway, also act sequentially to orchestrate resistance to ECTV infection.

## Ethics Statement

All mice were handled in accordance with the Good Animal Practice Requirements of the Animal Ethics Procedures and Guidelines of the People’s Republic of China, and the protocol was reviewed and approved by the Animal Ethics Committee of Lanzhou Veterinary Research Institute, Chinese Academy of Agricultural Science (Permit No. LVRIAEC2016-005).

## Author Contributions

W-YC, Z-ZJ, and X-BH conceived and designed the study and critically revised the manuscript. W-YC, H-JJ, G-HC, Z-LL, and Q-WJ performed the experiments, analyzed the data, and drafted the manuscript. W-YC wrote the paper. All the authors read and approved the final manuscript.

## Conflict of Interest Statement

The authors declare that the research was conducted in the absence of any commercial or financial relationships that could be construed as a potential conflict of interest.

## Supplementary Material

Figure S1 Ectromelia virus (ECTV) induces type I IFN production in L929 and RAW264.7 cells. **(A)** NIH3T3, L929, and RAW264.7 cells were either stimulated with ISD (at a final concentration of 1 µg/mL) or infected with ECTV at an MOI of 5. Supernatants were collected at 18 h post-infection (hpi). The concentrations of IFN-β in supernatants were determined by enzyme-linked immunosorbent assay (ELISA). RAW264.7 cells (1 × 10^6^) were infected with ECTV at an MOI of 0.1, 0.5, 1, 2, or 5. Subsequently, cells were washed twice with cold phosphate-buffered saline and were collected at 18 hpi. Total RNA was extracted using TRIzol reagent and reverse transcribed into cDNA, which was used to determine the relative mRNA levels of IFN-α **(B)** and IFN-β **(C)**. RAW264.7 cells (1 × 10^6^) were infected with ECTV at an MOI of 5. Cells and supernatants were separately collected at 0, 4, 8, 12, 15, 18, and 24 hpi. Total RNA was extracted using TRIzol Reagent and reverse transcribed into cDNA, which was determined the relative mRNA levels of IFN-α **(D)** and IFN-β **(E)**. The concentrations of IFN-β in supernatants were determined by ELISA **(F)**. All results are shown as mean ± SD from four independent experiments. Statistical analyses were performed by one-way analysis of variance followed by the Duncan’s multiple range test. In this figure, ND, not detected.

Figure S2 Sting and cGas are not required for the induction of IFN-β during ectromelia virus (ECTV) infection in NIH3T3 cells. **(A)** Western blot analysis of overexpressed cGas, Sting, and β-actin protein levels in NIH3T3 cells. **(B)** NIH3T3 (1 × 10^5^) cells were seeded in a 12-well plate and then were transfected with cGas, Sting-wt (S-wt), Sting-gt (S-gt), or empty (Vec.) plasmids. Thirty hours after transfection, cells were infected with ECTV (MOI of 5) for 18 h, and then the mRNA levels of IFN-α, IFN-β, and Ifit1 were analyzed by qPCR. **(C)** Western blot analysis of siRNA knockdown of cGas, Sting, and β-actin protein levels in NIH3T3 cells. **(D)** NIH3T3 (1 × 10^5^) cells were seeded in a 12-well plate and then were transfected with siRNAs for cGas (si-C1 and si-C2), Sting (si-S), or si-NC (si-N). Thirty-six hours after transfection, cells were infected with ECTV (MOI of 5) for 18 h, and then the mRNA levels of IFN-α, IFN-β, and Ifit1 were analyzed by qPCR. All the data represent mean ± SD of biological triplicates from at least three independent experiments. Statistical analyses were performed by one-way analysis of variance followed by the Duncan’s multiple range test. Con. means control group, which cells were only infected with ECTV (MOI of 5).

Figure S3 Ectromelia virus (ECTV) infection induces the phosphorylation of Tbk1 and Irf3 in L929 cells and RAW264.7 cells, but not in NIH3T3 cells. L929 cells **(A)** and NIH3T3 cells **(C)** (1 × 10^6^) were uninfected or infected with ECTV at an MOI of 5 and were collected at 4, 8, 12, 15, 18, and 24 h post-infection (hpi). **(B)** RAW264.7 cells (1 × 10^6^) were uninfected or infected with ECTV at an MOI of 5 and were collected at 3, 6, 12, 18, and 24 hpi. Results shown are representative of three independent experiments.
