# Supplementary figures and images for: The cGas–Sting Signaling Pathway Is Required for the Innate Immune Response Against Ectromelia Virus
Source: Front Immunol. 2018 Jun 14;9:1297. doi: 10.3389/fimmu.2018.01297 (PMC6010520; doi:10.3389/fimmu.2018.01297)

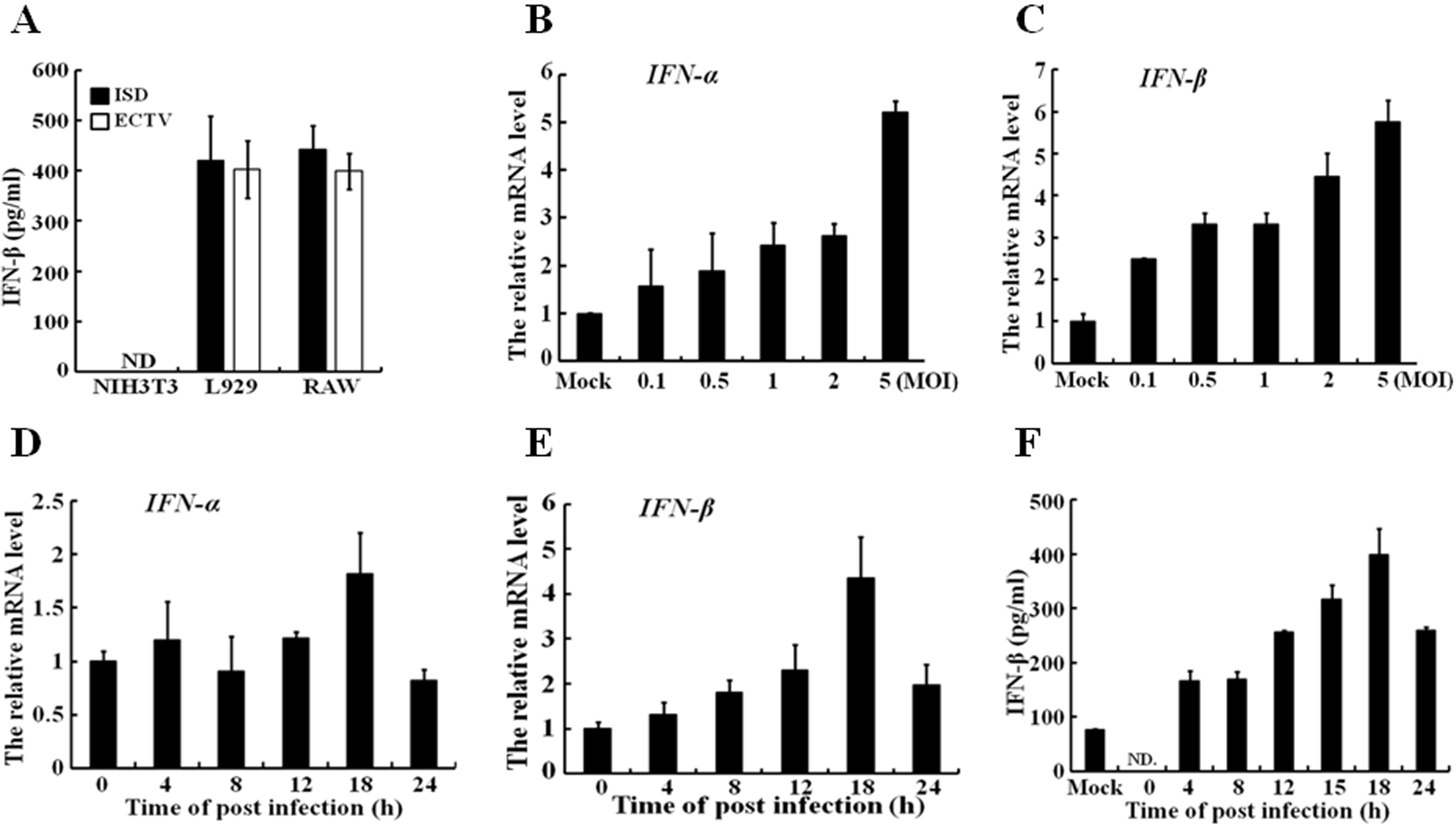

Supplement: Figure S1 — Ectromelia virus (ECTV) induces type I IFN production in L929 and RAW264.7 cells. (A) NIH3T3, L929, and RAW264.7 cells were either stimulated with ISD (at a final concentration of 1 µg/mL) or infected with ECTV at an MOI of 5. Supernatants were collected at 18 h post-infection (hpi). The concentrations of IFN-β in supernatants were determined by enzyme-linked immunosorbent assay (ELISA). RAW264.7 cells (1 × 10^6^) were infected with ECTV at an MOI of 0.1, 0.5, 1, 2, or 5. Subsequently, cells were washed twice with cold phosphate-buffered saline and were collected at 18 hpi. Total RNA was extracted using TRIzol reagent and reverse transcribed into cDNA, which was used to determine the relative mRNA levels of IFN-α (B) and IFN-β (C). RAW264.7 cells (1 × 10^6^) were infected with ECTV at an MOI of 5. Cells and supernatants were separately collected at 0, 4, 8, 12, 15, 18, and 24 hpi. Total RNA was extracted using TRIzol Reagent and reverse transcribed into cDNA, which was determined the relative mRNA levels of IFN-α (D) and IFN-β (E). The concentrations of IFN-β in supernatants were determined by ELISA (F). All results are shown as mean ± SD from four independent experiments. Statistical analyses were performed by one-way analysis of variance followed by the Duncan’s multiple range test. In this figure, ND, not detected. [file image_1.tif]

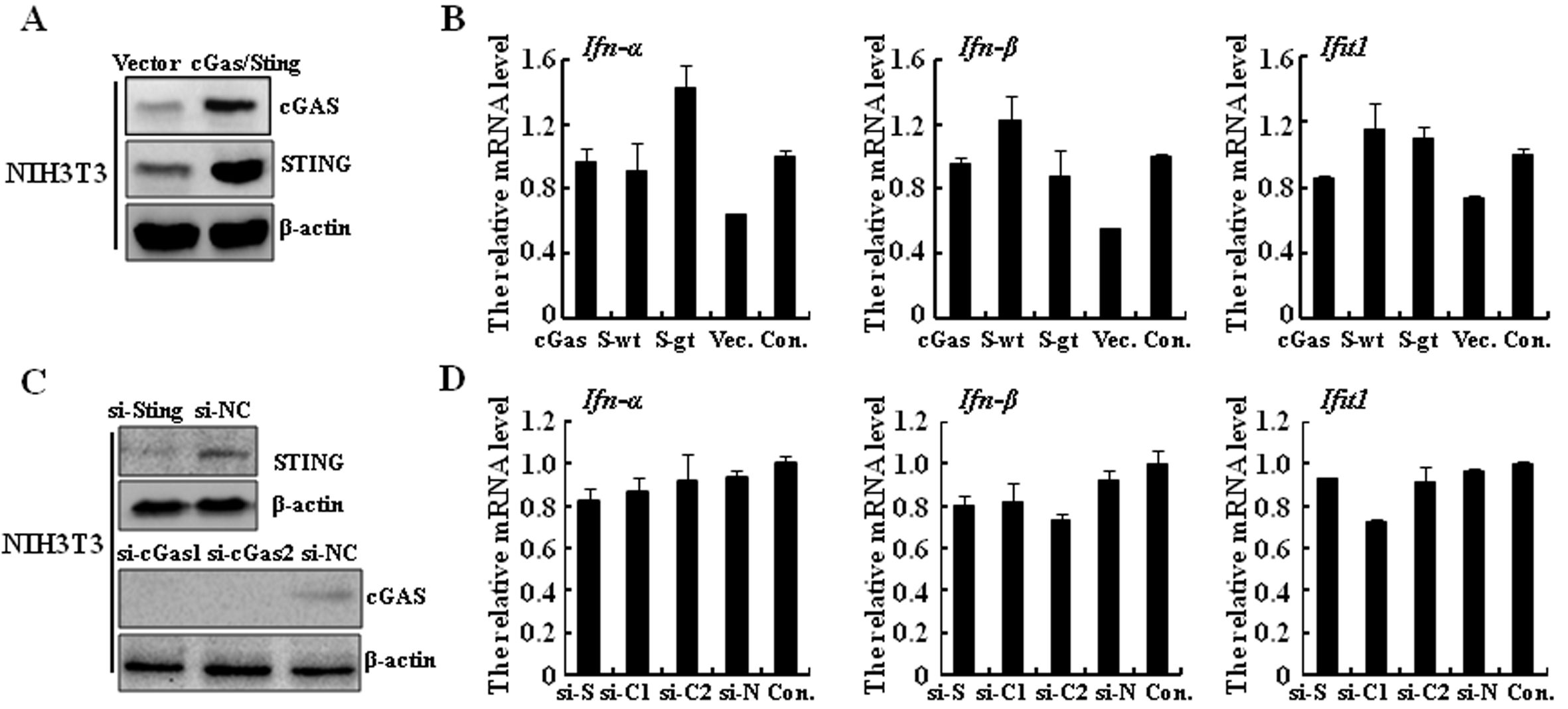

Supplement: Figure S2 — Sting and cGas are not required for the induction of IFN-β during ectromelia virus (ECTV) infection in NIH3T3 cells. (A) Western blot analysis of overexpressed cGas, Sting, and β-actin protein levels in NIH3T3 cells. (B) NIH3T3 (1 × 10^5^) cells were seeded in a 12-well plate and then were transfected with cGas, Sting-wt (S-wt), Sting-gt (S-gt), or empty (Vec.) plasmids. Thirty hours after transfection, cells were infected with ECTV (MOI of 5) for 18 h, and then the mRNA levels of IFN-α, IFN-β, and Ifit1 were analyzed by qPCR. (C) Western blot analysis of siRNA knockdown of cGas, Sting, and β-actin protein levels in NIH3T3 cells. (D) NIH3T3 (1 × 10^5^) cells were seeded in a 12-well plate and then were transfected with siRNAs for cGas (si-C1 and si-C2), Sting (si-S), or si-NC (si-N). Thirty-six hours after transfection, cells were infected with ECTV (MOI of 5) for 18 h, and then the mRNA levels of IFN-α, IFN-β, and Ifit1 were analyzed by qPCR. All the data represent mean ± SD of biological triplicates from at least three independent experiments. Statistical analyses were performed by one-way analysis of variance followed by the Duncan’s multiple range test. Con. means control group, which cells were only infected with ECTV (MOI of 5). [file image_2.tif]

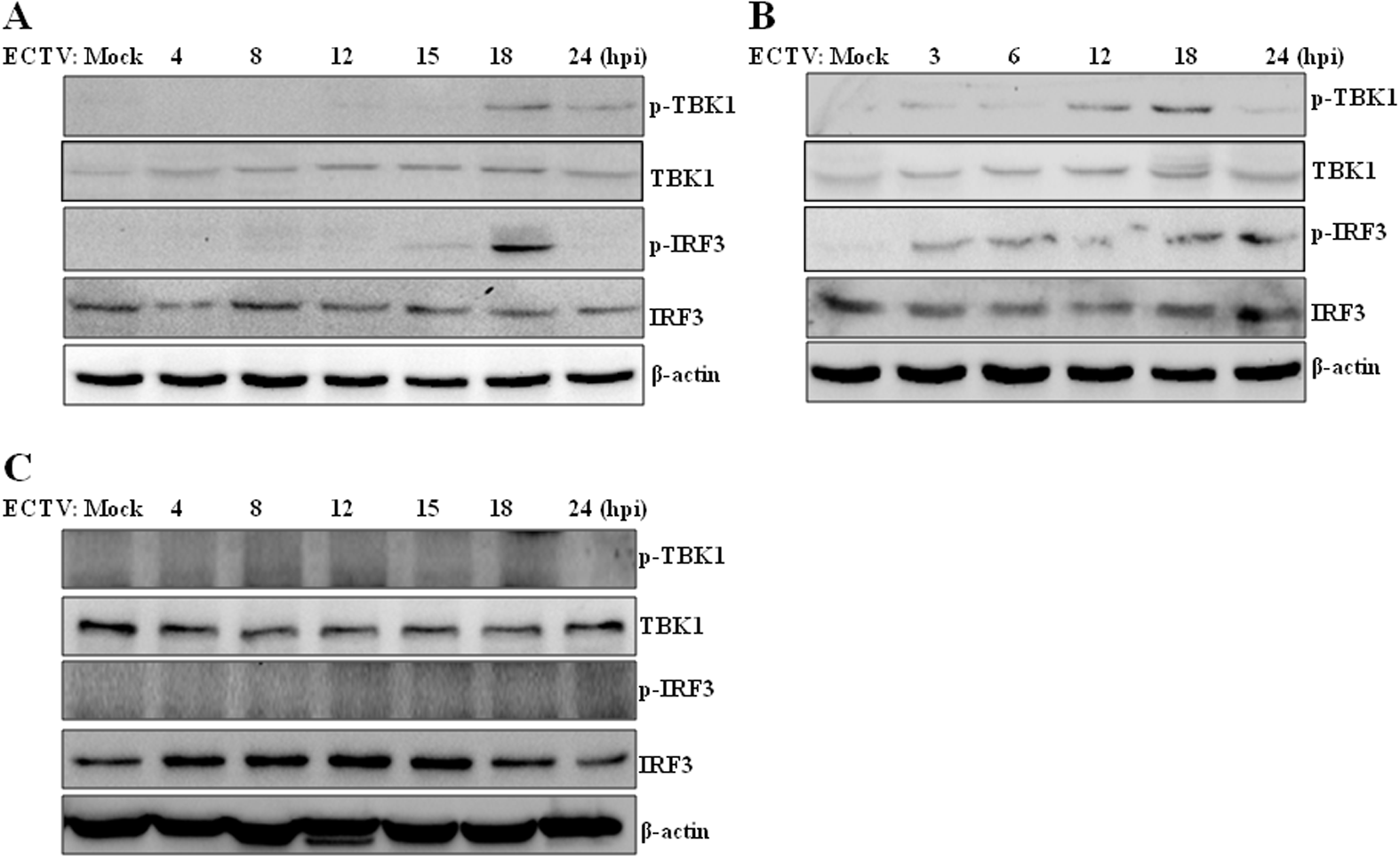

Supplement: Figure S3 — Ectromelia virus (ECTV) infection induces the phosphorylation of Tbk1 and Irf3 in L929 cells and RAW264.7 cells, but not in NIH3T3 cells. L929 cells (A) and NIH3T3 cells (C) (1 × 10^6^) were uninfected or infected with ECTV at an MOI of 5 and were collected at 4, 8, 12, 15, 18, and 24 h post-infection (hpi). (B) RAW264.7 cells (1 × 10^6^) were uninfected or infected with ECTV at an MOI of 5 and were collected at 3, 6, 12, 18, and 24 hpi. Results shown are representative of three independent experiments. [file image_3.tif]
